# Gender Differences in the Association between Lipid Profile and Sexual Function among Patients with Coronary Artery Disease

**Published:** 2014-01-01

**Authors:** Shervin Assari, Khodabakhsh Ahmadi, Davoud Kazemi Saleh

**Affiliations:** 1 Department of Health Behavior and Health Education, School of Public Health, University of Michigan, Ann Arbor, USA; 2 Center for Research on Ethnicity, Culture and Health, School of Public Health, University of Michigan, Ann Arbor, USA; 3 Behavioral Sciences Research Center, Baqiyatallah University of Medical Sciences, Tehran, IR Iran; 4 Department of Internal Medicine, Baqiyatallah University of Medical Sciences, Tehran, IR Iran

**Keywords:** Coronary Artery Disease, Gender, Sexual Behavior, Hyperlipidemias

## Abstract

**Background:**

Although several studies have been conducted on the association between lipid profile and sexual function among men with coronary artery disease, there is a paucity of knowledge about this association among women with coronary artery disease.

**Objectives:**

Our study aimed to evaluate the link between lipid profile and sexual function in men and women with coronary artery disease.

**Methods:**

One hundred and twenty patients with documented coronary artery disease were consecutively sampled from an outpatient cardiovascular clinic. The patients were assessed for lipid profile and sexual relationship using the Relation and Sexuality Scale (RSS). In addition, the Hospital Anxiety and Depression Scale (HADS) was used to measure the symptoms of anxiety and depression. The characteristics of chest pain were also measured using the Rose Angina Questionnaire. The data were analyzed through linear regression analysis.

**Results:**

This study was conducted on 91 males (75.8%) and 29 females (24.2%). Multivariate analysis showed that low-density lipoprotein cholesterol was correlated with sexual function (B = 0.01, P = 0.010) and total sexual relationship (B = 0.01, P = 0.050). A correlation was also observed between the level of high-density lipoprotein and sexual frequency score (B = -0.02, P = 0.040). Gender moderated these correlations. Among males, serum cholesterol (r = 0.193, P = 0.047) and low-density lipoprotein (r = 0.224, P = 0.037) were correlated to sexual function. In females, however, low-density lipoprotein was correlated to the total sexual relationship (r = 0.426, P = 0.021) and high-density lipoprotein was correlated to sexual frequency (r = -0.334, P = 0.046).

**Conclusions:**

The findings of this study showed a relationship between lipid profile and sexual relationship among both male and female patients with coronary artery disease. The link between lipid profile and sexual function of the patients with coronary artery disease is thus beyond just the effect of lipid profile on erectile dysfunction.

## 1. Background

Coronary artery disease and sexual dysfunction are both prevalent and tend to be comorbid (1). Sexual activity is an important component of well-being (2) and is a major concern to both the patients with coronary artery disease and their health care providers. These patients are often fearful of triggering Myocardial Infarction (MI) during intercourse and, as a result, may avoid sexual relations (3). On the other hand, vascular disorders may be presented as sexual dysfunction (4). Thus, it is reasonable to pay more attention to sexual relationships of patients with coronary artery disease.

Increased low-density lipoprotein and decreased high-density lipoprotein are both risk factors of coronary artery disease (5-7). Lipid disorders also influence sexual function in the general population (8-11). Unfortunately, there is a paucity of knowledge about sexual function in general and lipid profile. Generally, sexual function is influenced by a complex series of physical and mental factors that may change in patients with coronary artery disease.

We hypothesized that hyperlipidemia is associated with sexual function in both males and females suffering from coronary artery disease. Consequently, we assessed the relationship between lipid profile and sexual function, sexual frequency, and sexual fear among patients with coronary artery disease, with gender conceptualized as a moderator.

## 2. Materials and Methods

This cross-sectional study was conducted at an outpatient clinic in Tehran, Iran between June and December 2006. The study was approved by the local ethical committee of a medical university in Tehran, Iran. Written informed consents were also obtained from all the study patients.

Among the 150 candidate patients who were interviewed, 30 patients did not sign the informed consents or were excluded due to ineligibility. Thus, 120 patients were enrolled into the current study. The patients were eligible if they had documented coronary artery disease defined by a stenosis greater than 50% in at least 1 major coronary artery confirmed by angiography. The exclusion criteria of the study were MI and hospitalization during the past 6 months.

The socio-demographic data included age, sex, family income, and education level. We also collected data on the angiographic findings as well as on the history of hypertension, cigarette smoking, and diabetes mellitus.

Blood samples were taken from the peripheral veins after an overnight fasting of 12 hours. Fasting Blood Glucose (FBG), serum levels of triglycerides, serum levels of cholesterol, and plasma levels of high-density lipoprotein were determined by enzymatic methods using bioMerieux kits (Konelab, Cergy Pontoise, France) and auto-analyzer (Olympus Optical Co, Sizuoka, Japan). In addition, low-density lipoprotein was calculated according to the Friedewald formula (12).

In this study, a translation of the Hospital Anxiety and Depression Scale (HADS) (13) was used, including 14 statements relevant to generalized anxiety (7 items) and depression (7 items). This translated version of the HADS has been reported to be acceptable to almost all patients, with a Cronbach's alpha coefficient of 0.78 for the anxiety subscale and 0.86 for the depression subscale (14). Each item in HADS has 4 possible answers with scores ranging scores zero to 3. The maximum score was 21 for each scale. The Zigmond and Snaith cut-off point of scores greater than 8 was considered for assessment of depression and anxiety (13). Total score was also calculated. The Cronbach's Alpha of total score was 0.815. The translated version of HADS has been frequently used (15-22).

To evaluate the patients’ sexual relationship, the 10-item Relation and Sexuality Scale (RSS) was used (23, 24). The RSS evaluates the sexual relationships of a patient in comparison to that before the onset of the disease. Four or five simple Likert scales were used for each item and higher scores were indicative of worse conditions. In addition to the total score, 3 subscores, namely sexual function, sexual frequency, and sexual fear, were assessed, as well. Items 1, 2, 3, 7, and 8 were used for sexual function, items 4, 9, and 10 were used for sexual frequency, and items 5 and 6 were used for sexual fear. The Cronbach's Alpha was 0.802 for the total sexual relationship, 0.861 for sexual function, 0.820 for sexual frequency, and 0.769 for sexual fear. Translation of the RSS questionnaire was done in accordance with the guidelines for the Process of Translation and Adaptation of Instruments by taking the following steps: forward translation, back-translation, pretesting and cognitive interviewing, and developing the final version (25). The translated version of RSS has been frequently applied to patients with coronary artery disease and other chronic conditions (26-29).

For measurement of the characteristics of chest pain, the Rose Angina Questionnaire was used in this study (30). Rose angina grade I: The respondents reported pain that met the criteria for any exertional chest pain and that the pain caused them to stop or slow down, went away when the respondents stood still, disappeared within 10 minutes or less, occurred in the sternum, left arm and / or left anterior of the chest, and did not occur when walking at an ordinary pace on level ground. Rose angina grade II: The respondents reported pain that met the criteria for grade I Rose angina and reported that the pain occurred when walking at an ordinary pace on level ground.

All of the statistical analyses were performed using the SPSS statistical software (Statistical Package for Social Sciences, version 13.0, SPSS Inc., Chicago, IL, USA). The data were expressed as mean ± standard deviation or percentage where appropriate. The Kolmogorov-Smirnov test was used to check the normal distribution of the quantitative data while the Pearson bivariate correlation test was used for correlation analysis of the data. Spearman's rho correlation test was also employed to evaluate the correlation between lipid profile and sexual relationship for each gender group. Moreover, multivariate linear regression was used to evaluate the possible effects of other variables on the strength of the association between lipid profile and sexual function. The variables that were included in the model were age, sex, cigarette smoking, hypertension, diabetes, anxiety, depression, and angiographic and Rose Angina findings. P values of less than 0.05 were considered as statistically significant.

## 3. Results

This study was conducted on 120 patients. Demographic and clinical characteristics of the participants are outlined in Table 1. The medications used for these patients were as follows: 18.2% Benzodiazepins, 18.2% antidepressant, 47.6% beta blocker, 98.6% antiplatelet, 96.7% nitrate, and 89.7% calcium channel blockers.

**Table 1.** Demographic and Clinical Characteristics of the Patients with Coronary Artery Disease

Characteristics	Values
**Mean age (range), y**	57.11 ± 11.2 (36 - 79)
**Gender male**	91 (75.8)
**Female**	29 (24.2)
**Education illiterate**	26 (21.7)
**Primary school**	50 (41.7)
**Diploma**	23 (19.2)
**University**	21 (17.5)
**Family income, US$**	
**< 200**	33 (27.5)
**200 - 300**	57 (47.5)
**> 300**	30 (25.0)
**Cigarette smoking**	
**Yes**	22 (18.4)
**No**	98 (81.6)
**Hypertension**	
**Yes**	46 (38.3)
**No**	74 (61.7)
**Anxiety (0 - 21)**	6.9 ± 4.99
**Depression (0 - 21)**	5.9 ± 3.4
**Rose angina questionnaire no chest pain**	14 (11.6%)
**Non exertion chest pain**	4 (3.0%)
**Angina**	39 (32.5%)

The mean serum levels of triglycerides and cholesterol were 114.3 ± 42.6 mg / dL and 39.8 ± 14.1 mg / dL, respectively. In addition, the mean plasma levels of low-density lipoprotein and high-density lipoprotein cholesterol were 168.0 ± 80.0 mg / dL and 182.0 ± 58.5 mg / dL, respectively.

The mean total sexual function score was 16.6 ± 4.7 (range, 7 to 31). Additionally, mean scores of 10.3 ± 3.3 (range, 4 to 16), 5.5 ± 2.1 (range, 0 to 12), and 16.6 ± 4.7 (range, 7 to 31) were obtained for sexual function, sexual frequency, and sexual fear, respectively.

Moreover, the results revealed a correlation between the anxiety symptoms and HDL (P = 0.44, r = 0.286) and sexual frequency (P = 0.23, r = 0.207). The correlations between the RSS scores and lipid profile are shown in Table 2. The patients’ plasma levels of high-density lipoprotein were significantly correlated to their sexual frequency scores. Also, the plasma levels of low-density lipoprotein were significantly correlated to the sexual function and total sexual relationship. Cholesterol levels had a significant correlation with sexual function and total sexual relationship. As anticipated, sexual fear scores were not associated with the lipid profile. Also, no correlation was observed between triglycerides and sexual domains. In multivariate regression analyses (Table 3), total sexual relationship was only correlated to low-density lipoprotein among the lipid profile indicators and female gender was another influencing covariate. Moreover, sexual function was only associated with low-density lipoprotein and male gender. In a regression analysis with sexual frequency as outcome, the level of high-density lipoprotein was the only lipid profile indicator associated with the outcome. Female gender was another significant predictor.

**Table 2.** The Correlation between Lipid Profile and Sexual Function

		Total Sexual Relationship	Sexual Function	Sexual Frequency	Sexual Fear
**Cholesterol**	Pearson	0.162	0.185	0.044	0.02
	P value	0.047	0.043	0.63	0.82
**Triglyceride**	Pearson	0.03	0.036	0.095	0.06
	P value	0.73	0.69	0.30	0.51
**High-density cholesterol lipoprotein**	Pearson	0.031	-0.01	0.184	0.01
	P value	0.74	0.86	0.048	0.96
**Low-density cholesterol lipoprotein**	Pearson	-0.21	-0.23	0.01	-0.03
	P value	0.02	0.012	0.96	0.75

**Table 3.** Regression Analysis to Determine Predictors of Sexual Function among Men and Women with Coronary Artery Disease

Total Sexual Relationship			
	B	S.E	Sig
**Low-density lipoprotein cholesterol**	0.019	0.010	0.047
**Age**	0.77	0.182	0.048
**Sex**	2.829	0.970	0.004
**Constant**	10.881	3.014	0.000
**Model significance**			0.002
**Sexual function**			
**LDL**	0.018	0.007	0.011
**Age**	0.055	0.027	0.039
**Sex**	1.680	0.673	0.014
**Constant**	7.177	2.092	0.001
**Model significance**			0.001
**Sexual frequency**			
**Low-density lipoprotein cholesterol**	-0.023	0.013	0.048
**Anxiety**	0.097	0.043	0.050
**Sex**	1.600	0.434	0.000
**Constant**	0.739	1.271	0.562
**Model significance**			< 0.001

It should be noted that we analyzed the relations in each gender separately and found that the levels of total cholesterol and low-density lipoprotein cholesterol were significantly correlated to sexual function in males. In females, on the other hand, low-density lipoprotein was correlated to total sexual relationship and HDL cholesterol was correlated to sexual frequency (Table 4). 

**Table 4.** Correlation between Lipid Profile and Sexual Function among Men and Women with Coronary Artery Disease

		Total Sexual Relationship	Sexual Function	Sexual Frequency	Sexual Fear
**Men (N = 91)**					
**Total cholesterol**	Spearman	0.152	0.193	0.028	0.059
	P value	0.151	0.047	0.789	0.578
**Triglyceride**	Spearman	0.024	0.016	0.091	0.006
	P value	0.822	0.878	0.392	0.957
**High-density lipoprotein cholesterol**	Spearman	-0.020	-0.049	-0.128	-0.048
	P value	0.858	0.649	0.238	0.656
**Low-density lipoprotein cholesterol**	Spearman	0.168	0.224	0.034	0.047
	P value	0.121	0.037	0.756	0.667
**Women (N = 29)**					
**Total cholesterol**	Spearman	0.249	0.160	0.068	0.112
	P value	0.192	0.408	0.725	0.564
**Triglyceride**	Spearman	0.129	0.064	0.162	0.177
	P value	0.503	0.740	0.402	0.357
**High-density lipoprotein cholesterol**	Spearman	-0.159	-0.041	-0.334	-0.047
	P value	0.409	0.831	0.046	0.810
**Low-density lipoprotein cholesterol**	Spearman	0.426	0.265	0.130	0.103
	P value	0.021	0.164	0.502	0.593

## 4. Discussion

The findings of the present study indicated a relationship between lipid profile and different aspects of sexual function among males and females with coronary artery disease. Epidemiological studies in general populations and experimental investigations in animal models have indicated a close association between hyperlipidemia and sexual dysfunction (8, 10, 11). However, the role of plasma lipid levels in sexual dysfunction in general has been poorly described (31-33). Our findings are not restricted to erectile dysfunction, as sexual function and sexual frequency were evaluated, as well. On the other hand, several studies have just focused on erectile dysfunction, which is manifested only in men. Wei and colleagues first reported the association between the serum cholesterol and HDL cholesterol levels and erectile dysfunction in males with manifestations of arteriosclerotic diseases (33). In a prospective review of 3,250 males aging from 26 to 83 years without erectile dysfunction at their first examination, total cholesterol and high-density lipoprotein levels were found to be strongly predictive of the onset of erectile dysfunction after controlling for age, diabetes mellitus, stress level, cardiovascular disease, and prostate disease (33).

Hyperlipidemia has been implicated in the development of sexual dysfunction by several different mechanisms (33). The first mechanism might be the impairment of endothelium-dependent relaxation in the smooth muscle cells of the corpus cavernous due to hypercholesterolemia (34). A reduction in nitric oxide production may be another explanation for development of sexual problems in patients with hypercholesterolemia. Several studies have shown that endothelial relaxation is impaired if the blood vessel wall is exposed to oxidized low-density lipoprotein; these free radicals can inactivate nitric oxide (35).

In men, penile vascular changes have been noted in impotent patients with elevated serum lipids (33). Almost all previous studies on the association between lipid profile and sexual function have been conducted on males. In this study, however, we found a relationship between lipid profile and sexual function both in males and females with coronary artery disease. Yet, different patterns of associations were seen in males and females with coronary artery disease. The predominant relation was between low-density lipoprotein and sexual function in males. In females, on the other hand, low-density lipoprotein was correlated to sexual relationship and high-density lipoprotein was correlated to sexual frequency. Therefore, the relationship between lipids and sexual function in coronary artery disease might be something more than only endothelial dysfunction.

Furthermore, anxiety was a predictor of sexual frequency in our patients. Anxiety tends to be comorbid with and might be a cause or consequence of coronary artery disease. Anxiety symptoms may change with the natural course of coronary artery disease and may be a consequence of the physical limitations in activities of daily living as well as fear of poor prognosis (36, 37).

Performance anxiety and fear of death also play a role in sexual dysfunction (38). Additionally, the condition of sexual dysfunction may play a role in the genesis of anxiety. Of course, these psychological conditions can be improved by successful sexual dysfunction treatment (39).

Previous studies have shown that hormones play a significant role in regulating female sexual function (40). Low testosterone levels are also associated with a decline in sexual arousal, genital sensation, libido, and orgasm. However, clinical studies are in the process of assessing the potential benefits of testosterone for treatment of female sexual dysfunction (41, 42). The interesting point that supports our findings in women is that testosterone significantly decreases serum lipid levels in women (43, 44). This point can explain the cause of concurrent impaired sexual function and lipid levels in the women with coronary artery disease in this study (45, 46). The frequency of sexual relations is a factor that can be influenced by many factors other than sexual function, such as arousal and libido. In contrast to males, the females in our study had intercourse less frequently if they had impaired low-density lipoprotein cholesterol and high-density lipoprotein. We can speculate that the effect of lipids on testosterone levels in women may impact their sexuality, which is a different mechanism from that in men with erectile dysfunction.

This study had some limitations. First of all, one-fifth of the patients with coronary artery disease refused to provide data on sexual function and were excluded from this study. It is quite likely that these patients were among those with greater sexual problems or those who had no significant sexual problems. Therefore, the study is prone to sampling bias. In addition, sex hormones, thyroid hormones, and metabolic syndromes that may interfere with sexual functioning were not included in our analysis. Although the presence of a correlation between lipid profile and sexual function in patients with coronary artery disease was found, illustrating their causal relationship is beyond the scope of this study. As one of the strengths of this study, we measured the patients’ sexual function using RSS. This measure is not confined to sexual dysfunctions. However, we acknowledge that a definite conclusion needs further research, especially with regard to the modest correlations that we herein reported.

Poor sexual function is being reported among men and women with coronary artery disease. Irrespective of gender, dyslipidemia should be considered as a risk-factor associated with impaired sexual function among patients with coronary artery disease. Thus, the effect of lipid profile on sexual function of patients with coronary artery disease is beyond the well-described effect of lipid disorders on erectile dysfunction among male patients.
